# Improvement of conformal arc plans by using deformable margin delineation method for stereotactic lung radiotherapy

**DOI:** 10.1002/acm2.12237

**Published:** 2017-12-07

**Authors:** Görkem Güngör, Melek Demir, Gökhan Aydın, Bülent Yapıcı, Banu Atalar, Enis Özyar

**Affiliations:** ^1^ Department of Radiation Oncology Acıbadem University School of Medicine Istanbul Turkey

**Keywords:** DCA, FFF, SBRT, VMAT

## Abstract

**Purpose:**

Stereotactic body radiotherapy (SBRT) is an established treatment technique in the management of medically inoperable early stage non–small cell lung cancer (NSCLC). Different techniques such as volumetric modulated arc (VMAT) and three‐dimensional conformal arc (DCA) can be used in SBRT. Previously, it has been shown that VMAT is superior to DCA technique in terms of plan evaluation parameters. However, DCA technique has several advantages such as ease of use and considerable shortening of the treatment time. DCA technique usually results in worse conformity which is not possible to ameliorate by inverse optimization. In this study, we aimed to analyze whether a simple method – deformable margin delineation (DMD) – improves the quality of the DCA technique, reaching similar results to VMAT in terms of plan evaluation parameters.

**Methods:**

Twenty stage I–II (T1‐2, N0, M0) NSCLC patients were included in this retrospective dosimetric study. Noncoplanar VMAT and conventional DCA plans were generated using 6 MV and 10 MV with flattening filter free (FFF) photon energies. The DCA plan with 6FFF was calculated and 95% of the PTV was covered by the prescription isodose line. Hot dose regions (receiving dose over 100% of prescription dose) outside PTV and cold dose regions (receiving dose under 100% of prescription dose) inside PTV were identified. A new PTV (PTV‐DMD) was delineated by deforming PTV margin with respect to hot and cold spot regions obtained from conventional DCA plans. Dynamic multileaf collimators (MLC) were set to PTV‐DMD beam eye view (BEV) positions and the new DCA plans (DCA‐DMD) with 6FFF were generated. Three‐dimensional (3D) dose calculations were computed for PTV‐DMD volume. However, the prescription isodose was specified and normalized to cover 95% volume of original PTV. Several conformity indices and lung doses were compared for different treatment techniques.

**Results:**

DCA‐DMD method significantly achieved a superior conformity index (CI), conformity number (CI_P__addick_), gradient index (R_50%_), isodose at 2 cm (D_2 cm_) and external index (CΔ) with respect to VMAT and conventional DCA plans (*P* < 0.05 for all comparisons). CI ranged between 1.00–1.07 (Mean: 1.02); 1.00–1.18 (Mean: 1.06); 1.01–1.23 (Mean 1.08); 1.03–1.29 (Mean: 1.15); 1.04–1.29 (Mean: 1.18) for DCA‐DMD‐6FFF, VMAT‐6FFF, VMAT‐10FFF DCA‐6FFF and DCA‐10FFF respectively. DCA‐DMD‐6FFF technique resulted significantly better CI compared to others (*P* = 0.002; < 0.001; < 0.001; < 0.001). R_50%_ ranged between 3.22–4.74 (Mean: 3.99); 3.24–5.92 (Mean: 4.15) for DCA‐DMD‐6FFF, VMAT‐6FFF, respectively. DCA‐DMD‐6FFF technique resulted lower intermediate dose spillage compared to VMAT‐6FFF, though the difference was statistically insignificant (*P* = 0.32). D_2 cm_ ranged between 35.7% and 67.0% (Mean: 53.2%); 42.1%–79.2% (Mean: 57.8%) for DCA‐DMD‐6FFF, VMAT‐6FFF respectively. DCA‐DMD‐6FFF have significantly better and sharp falloff gradient 2 cm away from PTV compared to VMAT‐6FFF (*P *= 0.009). CΔ ranged between 0.052 and 0.140 (Mean: 0.085); 0,056–0,311 (Mean: 0.120) for DCA‐DMD, VMAT‐6FFF, respectively. DCA‐DMD‐6FFF have significantly improved CΔ (P = 0.002). VMAT‐ V_20 Gy_, V_2.5 Gy_ and mean lung dose (MLD) indices are calculated to be 4.03%, 23.83%, 3.42 Gy and 4.19%, 27.88%,3.72 Gy, for DCA‐DMD‐6FFF and DCA techniques, respectively. DCA‐DMD‐6FFF achieved superior lung sparing compared to DCA technique. DCA‐DMD‐6FFF method reduced MUs 44% and 33% with respect to VMAT‐6FFF and 10FFF, respectively, without sacrificing dose conformity (*P* < 0.001; *P* < 0.001).

**Conclusions:**

Our results demonstrated that DCA plan evaluation parameters can be ameliorated by using the DMD method. This new method improves DCA plan quality and reaches similar results with VMAT in terms of dosimetric parameters. We believe that DCA‐DMD is a simple and effective technique for SBRT and can be preferred due to shorter treatment and planning time.

## 1 INTRODUCTION

SBRT is the delivery of a curative radiation dose to a visible gross tumor in a very precise way, using image guidance generally in 1 to 5 fractions.^1^, ^2^, ^3^, ^4^, ^5^, ^6^, ^7^ Early studies have shown that SBRT is an effective and well‐tolerated treatment for early stage inoperable non–small cell lung cancer (NSCLC) patients.^6^, ^7^, ^8^, ^9^ SBRT can be delivered with 4 different techniques; three dimensional conformal multiple static beams (3DC) with coplanar or noncoplanar fields, three‐dimensional conformal arc (DCA), intensity modulated radiotherapy (IMRT) and volumetric modulated arc therapy (VMAT). Each method has different advantages and disadvantages.

The DCA technique widely replaced 3DC techniques with its advantage of using large number of beam directions and shorter treatment time.^10^, ^11^ Moreover, DCA plans have better conformity in three‐dimensional complex target volume shapes, converging to quasi‐sphere form can result because of better DCA conformity than 3DC plans.^12^ Moreover, since the dynamic field shape encompasses the target volume, DCA can avoid interplay effect because of shorter delivery time and continuous dynamic field openings during treatment delivery.^12^ Despite the interplay effect concern of intrafractional target volume motion, coplanar and noncoplanar inversely optimized IMRT techniques are also used safely in SBRT treatments.^13^, ^14^, ^15^ However, it is largely replaced by VMAT due to the shorter treatment delivery time and improved target dose conformity.^16^, ^17^, ^18^ Recent removal of flattening filter from the beam generation module increased dose rates 2.5 to 4 times for different photons energies. This led to significant shortening of the treatment delivery time for both DCA and VMAT techniques.^19^, ^20^ FFF‐based techniques recently became a standard treatment for SBRT.^21^, ^22^, ^23^, ^24^, ^25^ It has also been shown that VMAT‐FFF has led to better conformity parameters with shorter treatment delivery time than 3DC, DCA, IMRT, and VMAT techniques.^22^, ^23^, ^24^, ^25^, ^26^, ^27^

There are similarities between DCA and VMAT techniques. Both techniques use arc method, and treatment times are significantly short. VMAT technique results in better conformity due to use of inverse optimization method during planning but with the cost of a longer time for planning process and quality assurance. However, it is easy to generate DCA plan but difficult to achieve high dose conformity for complex shaped target volumes compared to VMAT. Therefore, several authors have investigated different modifications of DCA technique to improve the dose distributions with modification of either dynamic conformal technique or planning target volume (PTV).^28^, ^29^, ^30^, ^31^, ^32^ In this study, we investigated a simple method to ameliorate the dose conformity in patients treated with DCA technique. If our new method leads to better dose evaluation parameters than conventional DCA and reaches similar results to VMAT technique, it may considerably decrease the treatment planning time, increase dose conformity favorably and decrease treatment time.

## 2 MATERIALS AND METHODS

This retrospective dosimetric study included a total of 20 stage I–II (T1‐2, N0, M0) NSCLC patients treated with SBRT in our department. Lesions were chosen to be representative of the most frequent type of stage I–II tumors diagnosed in the clinical setting. Therefore, the location, volume and size of the tumors investigated was heterogeneous (7 tumors are centrally and 13 tumors are peripherally located; 12 tumors are in the left and 8 tumors are in the right lung; 7 tumors are in the upper lobes and 13 tumors are in the lower lobes). Target volume and fractionation characteristics are summarized in Table 1.

**Table 1:** Target and fractionation characteristics

Characteristics	Value
ITV (cc)	
Mean	17.29
Median	7.35
Range	0.56–69.39
PTV (cc)	
Mean	41.01
Median	22.57
Range	4.59–123.49
Fractionation scheme (Gy)	
3 × 18	7 of 20
5 × 11	8 of 20
8 × 7.5	5 of 20

All patients underwent four dimensional CT (4DCT) (Biograph 16, Siemens Medical Solutions, Erlangen Germany) scans with wing board arms up without vacuum cushion or abdominal compression. By obtaining 4D‐CT, maximum‐intensity projection (MIP) image sets were created and used to help define the internal target volume (ITV). Furthermore, PTV margin was delineated with an isotropic 5 mm expansion of ITV. Average intensity projection CT (AveIP‐CT) was also created in order to perform three‐dimensional dose calculations. In addition, organ at risk (OAR), such as left and right lung, chest wall, trachea, spinal cord, esophagus, heart, and great vessels were delineated in AveIP‐CT.

### 2.A Treatment planning and features

For each patient, conventional DCA–FFF, VMAT‐FFF and DCA‐FFF with deformable margin delineation (DCA‐DMD) method plans were generated, utilizing 6 MV and 10 MV photon energies for a TrueBeam STx linear accelerator (Varian Medical Systems, Palo Alto, CA, USA). The prescription isodose lines covering the 95% volume of PTV (V_PTV_) were normalized to 70%–85% of isodose line. Several dose constraints for different fractionations were used for different OARs which are summarized in Table 2.^1^, ^33^, ^34^, ^35^, ^36^, ^37^

**Table 2:** OAR constraints for different fractionation schemes

OAR	Limit	3 fractions	5 fractions	8 fractions
Chest wall	V_30 Gy_	30 cc	30 cc	–
	V_60 Gy_	3 cc	3 cc	–
Esophagus	D_max_	25.2 Gy	35 Gy	40 Gy
	D_5 cc_	17.7 Gy	19.5 Gy	–
Great vessels	D_max_	45 Gy	53 Gy	53 Gy
	D_10 cc_	39 Gy	47 Gy	47 Gy
Heart/pericardium	D_max_	30 Gy	38 Gy	–
	D_15 cc_	24 Gy	32 Gy	–
Lungs	V_20 Gy_	10%	10%	10%
	V_2.5 Gy_	30%	30%	–
	MLD	10 Gy	10 Gy	–
Spinal cord	D_max_	21.9 Gy	30 Gy	30 Gy
	V_10%_	18 Gy	23 Gy	–
	V_0.35 cc_	18 Gy	23 Gy	–
	V_1.2 cc_	12.3 Gy	14.5 Gy	–
Trachea	D_max_	30 Gy	40 Gy	44 Gy

### 2.B Dynamic conformal arc (DCA)

Conventional DCA plans were generated, using 340° coplanar arcs in order to obtain acceptable coverage, field aperture size and shape corresponded identically to the projection of the PTV outline along the beam's eye view. Each control point was dynamically adjusted during arc rotation according to radiation therapy oncology group (RTOG) 0915 protocol guidelines.^33^ Eclipse treatment planning system (TPS) was used with analytical anisotropic algorithm (AAA) dose calculation (v. 13.6.2) based upon 6 MV and 10 MV energies with FFF modality.

### 2.C VMAT

The VMAT plans were created, using commercial RapidArc^®^ module in Eclipse™ TPS with progressive resolution optimization (PRO3) (v. 13.6.2) solution. The PRO3 module was mainly based on direct aperture optimization approach varying with multileaf collimators (MLC), gantry speed and dose rate on each control point (CP).^38^ The PRO3 module proceeded through four phases at the same time. The full collection of 178 CPs was optimized in all four phases while dose calculation was still in progress. Each VMAT plan consisted of two coplanar arcs with clockwise and counter‐clockwise rotation. Collimator rotation angles of arcs were 10° and 350°, respectively, with 0 mm MLC margin to the outline of PTV. Arc entrance through the contralateral healthy lung was restricted as much as possible. AAA (v 13.6.2) was used in order to obtain three‐dimensional dose distributions for evaluation of 6 MV and 10 MV with FFF plans.

### 2.D Dynamic conformal arc with deformable margin delineation (DCA‐DMD)

**Figure 1:**
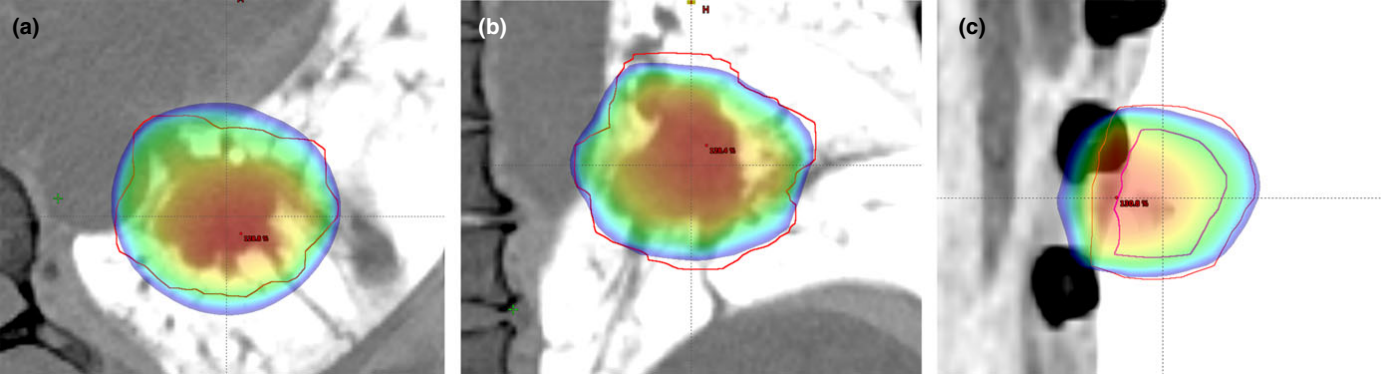
Dose conformity problems of the DCA technique. (a) High dose regions at anterior posterior (AP) direction. (b) High dose regions at left right (LR) and low dose regions at inferior superior (IS) directions. (c) Shift of high dose region out of ITV.

DCA plans usually result in nonconformal coverage. Undesired hot spot and cold spot dose regions around PTV and especially shift of high dose volume out of ITV are general problems for conventional DCA treatment plans (shown in Fig. 1).

Firstly, a conventional DCA plan with 6FFF was generated and 100% of prescription isodose line covering the 95% V_PTV_ was normalized. The prescription isodose lines were specified with covering the 95% volume of PTV (V_PTV_) which were normalized to 70%–85% of isodose.

We identified dose regions outside the PTV receiving doses over 100% of prescription dose (as hot spots) and inside the PTV receiving a dose under 100% of prescription dose (as cold spots). The new PTV, deformable margin delineated PTV (PTV‐DMD), was delineated with the help of deformation of PTV according to identified hot and cold spot dose regions, which were obtained from conventional DCA plan, toward either the negative or positive directions.

**Figure 2:**
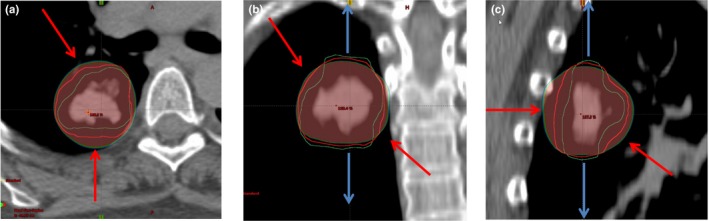
Propagation of new PTV (PTV‐DMD [Green]) by deforming original PTV [Red] according to hot and cold colorwash volumes. (a) Negative deformation from anterior direction (Red arrow). (b) Positive deformation margins from anterior and posterior directions (blue arrows). Negative deformation margins from left and right directions (red arrows). (c) Both negative and positive deformation margins from anterior/posterior/inferior directions (red and blue arrows). Red contour = original PTV, Green contour = PTV‐DMD.

If a hot spot region was present outside the PTV, PTV‐DMD was created by deformable shrinkage of PTV and if a cold spot region was present inside PTV, PTV‐DMD was created by deformable expansion of PTV (shown in Fig. 2).

Finally, a new DCA plan with 6FFF photon energy (DCA‐DMD plan) was generated, using PTV‐DMD volume. 3D dynamic MLC positions were set to beam eye view of PTV‐DMD and 3D dose calculations were computed for PTV‐DMD volume. However, the prescription isodose was normalized to cover 95% volume of original PTV after 3D dose calculation.

### 2.E Evaluation of treatment planning dosimetric parameters

Target conformity and organ at risk (OAR) doses were compared for DCA (6FFF‐10FFF), VMAT (6FFF‐10FFF) and DCA‐DMD (6FFF) planning techniques. Target conformity was evaluated by analyzing the following parameters: Dose to 2%, 50% and 98% volume of PTV (D_2%_, D_50%_ and D_98%_), maximum dose of PTV (D_max_), conformity index (CI),^33^ conformity Paddick index (CI_Paddick_),^39^ homogeneity index (HI) 40, 41 inside PTV, ratio of 50% isodose volume to PTV volume (R_50%_),^33^ maximum relative isodose at any point 2 cm or further away from PTV (D_2 cm_)^33^ and external index (CΔ).^42^ MU was considered for its treatment delivery efficiency. All plans were evaluated using RTOG 0915 prescription dose constraints for treatment planning guidelines and ICRU recommendations.^33^, ^40^, ^41^

### 2.F Conformity index (CI)

The RTOG conformity index is defined as ratio of prescription isodose volume (V_Rx_) to the PTV volume.^33^ Ideal value of CI is unity and generally it is greater than one.

(1) $$\mathrm{CI}=V_{\mathrm{Rx}}/V_{\mathrm{PTV}}$$

### 2.G Conformity Paddick index or conformity number (CI_Paddick_)

A new conformity index (CI_Paddick_) was proposed by Paddick^39^ as it does not produce false perfect scores.^43^ CI_Paddick_ denoted as

(2) $$CI_{\mathrm{Paddick}}=\frac{TV_{\mathrm{PIV}}}{\mathrm{TV}}\;*\;\frac{TV_{\mathrm{PIV}}}{\mathrm{PIV}}=\frac{{\left(TV_{\mathrm{PIV}}\right)}^{2}}{\mathrm{TV}*\mathrm{PIV}}$$

where TV, PIV are target volume and prescribed isodose volume, respectively, and TV_PIV_ is the volume of target covered by prescription isodose.^39^ CI_Paddick_ can be described as the multiplication of conformity and selectivity of plan. The first fraction of this equation defines the quality of coverage of target; the second fraction defines the volume of healthy tissue receiving a dose greater than or equal to the prescribed reference dose.^44^ Ideal value of CI_Paddick_ is unity and generally less than one.

### 2.H Gradient index (GI)

The ratio of 50% prescription isodose volume to the PTV volume is R_50%_.^33^ None and only minor deviations of R_50%_ were accepted while evaluating plans with respect to the PTV volume for SBRT plans according to RTOG 0915 protocol guidelines. Where this index value falls below none deviation, it refers to a sharp dose fall off around intermediate dose spillage region. Paddick I. and Lippitz B. proposed to use gradient index (GI) for SRS plans in 2006^43^

(3) $$R_{50\%}=\frac{V_{50\%}}{V_{\mathrm{PTV}}}$$

### 2.I Intermediate dose spillage location at 2 cm (D_2 cm_)

Intermediate dose spillage location is defined as the maximum dose in percentage of dose prescribed at 2 cm away from PTV in any direction (D_2 cm_).^33^

### 2.J Homogeneity index (HI)

The dose homogeneity of PTV,^41^ is described as

(4) $$HI=\;\frac{\left(D_{2\%}-D_{98\%}\right)}{D_{50\%}}$$

where D_2%_, D_50%,_ and D_98%_ are the dose values by 2%, 50% and 98% volume of PTV, respectively

### 2.K External index (EI)

The external index describes the exposure ratio of health tissue,^35^, ^42^ described as:

(5) $$C\Delta =\frac{\left(V_{\mathrm{PI}}-\mathrm{PT}V_{\mathrm{PI}}\right)}{\mathrm{PTV}},$$

where PI is prescription isodose, V_PI_ denotes total tissue volume received prescribed dose and PTV_PI_ denotes planning target volume received prescribed dose.

### 2.L Organs at risk dosimetric evaluation

Volume of 20 Gy, 2.5 Gy and mean dose of lungs (V_20_, V_2.5_, and D_mean_) were investigated. As previously described, the tumors investigated were located in different regions (central, peripheral, different lobes, etc.). The OARs for each lesion differed due to location thus data related to OARs other than lung was insufficient to make comparison between different planning techniques. Since random patients and target locations were chosen, it was statistically insignificant to collect plan acceptance data for spinal cord, heart, esophagus, trachea, chest wall, bronchus, and great vessels to compare**,** but dose constraints were used in optimization of VMAT plans to achieve acceptable OAR's limits.

### 2.M Statistical analysis

The Shapiro test was used to check whether the parameters were normally distributed. Wilcoxon rank test was performed to compare the dosimetric parameters of DCA, VMAT and DCA with DMD techniques. The tests were statistically significant if *P* value was < 0.05. The errors indicated interpatients’ variability at 1 standard deviation level. Correlation analysis was applied with Spearman Correlation analysis, using SPSS 23 (IBM, New York, NY, USA).

## 3 RESULTS

**Figure 3:**
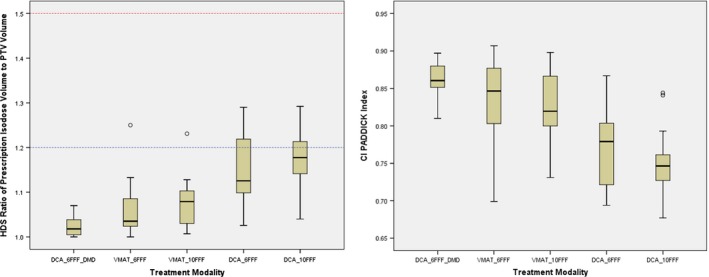
CI and CI_P__addick_ values for all techniques. Red and blue dashed lines refer to minor and none deviation values of 1.5 and 1.2, respectively.

The comparison of mean dosimetric evaluation parameters for DCA‐DMD‐6FFF, VMAT‐6FFF, VMAT‐10FFF, DCA‐6FFF and DCA‐10FFF are presented in Table 3. All techniques were compared according to mean CI and CI_Paddick_ , values, and it was observed that DCA‐DMD‐6FFF plans demonstrated the best mean CI (1.02 ± 0.02) and CI_Paddick_ (0.861 ± 0.02) values, respectively, compared to VMAT‐6FFF (1.06 ± 0.06 and 0.835 ± 0.05), VMAT‐10FFF (1.08 ± 0.05 and 0.823 ± 0.05), conventional DCA‐6FFF (1.15 ± 0.07 and 0.771 ± 0.05) and DCA‐10 FFF (1.18 ± 0.07 and 0.735 ± 0.04) plans. A box plot representation of these finding is shown in Fig. 3. It was observed that both conformity parameters for DCA‐DMD‐6FFF were statistically significantly compared to other techniques (*P* = 0.002; < 0.001; < 0.001; < 0.001 for CI and *P* = 0.01; = 0.02; < 0.001; < 0.001 for CI_Paddick_).

**Table 3:** The dosimetric evaluation results for different techniques. Dosimetric results are the mean of indices ± one standard deviation (SD) for 20 patients

Parameter	DCA‐DMD‐6FFF	VMAT‐6FFF	VMAT‐10FFF	DCA‐6FFF	DCA‐10FFF	*P* value (DCA‐DMD‐6FFF vs. others respectively)
PTV D_mean_ (%)	116.3 ± 3.81	115.6 ± 5.13	117.1 ± 4.68	120.7 ± 4.01	122.8 ± 3.42	*P* = 0.60; < 0.01; < 0.001; < 0.001
PTV D_max_ (%)	131.7 ± 9.00	132.9 ± 9.01	134.8 ± 8.25	135.9 ± 8.40	141.5 ± 6.04	*P* = 0.13; = 0.02; < 0.001; < 0.001
PTV D_2%_ (%)	129.7 ± 7.89	128.2 ± 7.55	131.4 ± 7.42	134.1 ± 7.47	139.3 ± 5.69	*P* = 0.06; = 0.01; < 0.001; < 0.001
PTV D_98%_ (%)	96.5 ± 0.99	96.0 ± 3.91	96.1 ± 3.60	93.5 ± 2.66	93.5 ± 2.20	*P* = 0.60; = 0.60; < 0.001; < 0.001
CI	1.020 ± 0.02	1.060 ± 0.06	1.080 ± 0.05	1.150 ± 0.07	1.180 ± 0.07	*P* = 0.002; < 0.001; < 0.001; < 0.001
CI_Paddick_	0.861 ± 0.02	0.835 ± 0.05	0.823 ± 0.05	0.771 ± 0.05	0.735 ± 0.04	*P* = 0.01; = 0.02; < 0.001; < 0.001
R_50%_	3.99 ± 0.40	4.15 ± 0.58	4.32 ± 0.66	4.17 ± 0.52	4.40 ± 0.52	*P* = 0.32; = 0.004; = 0.04; < 0.001
D_2 cm_ (%)	53.23 ± 9.97	57.80 ± 10.83	56.91 ± 10.44	57.35 ± 10.14	58.06 ± 10.77	*P* = 0.009; = 0.01; < 0.001; < 0.001
HI	0.284 ± 0.06	0.276 ± 0.07	0.299 ± 0.06	0.334 ± 0.07	0.371 ± 0.05	*P* = 0.57; = 0.46; = 0.001; < 0.001
CΔ	0.085 ± 0.02	0.120 ± 0.06	0.135 ± 0.05	0.207 ± 0.07	0.238 ± 0.06	*P* = 0.002; = 0.001; = 0.001; < 0.001
MU	2254 ± 860	3250 ± 1029	2994 ± 923	2323 ± 897	2132 ± 800	*P* < 0.001; < 0.001; = 0.014; = 0.002
Lung V_20 Gy_ (%)	4.19 ± 3.33	4.03 ± 3.56	4.26 ± 3.63	4.36 ± 3.07	4.46 ± 3.27	*P* = 0.88; = 0.062; = 0.079; = 0.086
Lung V_2.5 Gy_ (%)	27.88 ± 15.12	23.83 ± 13.46	25.73 ± 14.21	26.23 ± 14.07	27.41 ± 14.36	*P* < 0.001; < 0.001; = 0.03; = 0.04
MLD (Gy)	3.72 ± 2.28	3.42 ± 2.03	3.52 ± 2.08	3.59 ± 2.14	3.72 ± 2.25	*P* < 0.001; = 0.020; = 0.03; = 0.827

**Figure 4:**
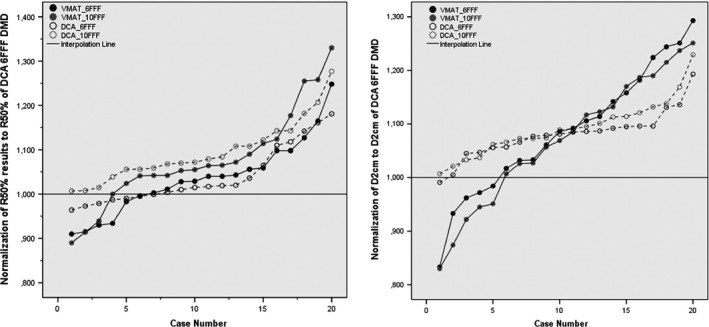
Normalized Graphs of R_50%_ and D_2 cm_. All R_50%_ and D_2 cm_ results of other techniques are normalized to the values of DCA 6FFF DMD technique. (a) Normalized R_50%_ (b) Normalized D_2 cm_. Solid line passes from 1.000 which is 3DCA 6FFF DMD.

Intermediate dose spillage parameters (R_50%_, D_2 cm_) are also compared for all techniques. Among all, DCA‐DMD‐6FFF plans achieved the best R_50%_ and D_2 cm_ (3.99% and 53.23%**,** respectively) values with respect to VMAT‐6FFF (4.15% and 57.80%), VMAT‐10FFF (4.32% and 56.91%), conventional DCA‐6FFF (4.17% and 57.35%) and DCA‐10 FFF (4.40% and 58.06%) plans respectively. R_50%_ and D_2 cm_ values were found to be similar to DCA‐DMD‐6FFF and VMAT‐6FFF techniques. (*P* = 0.32) However, DCA‐DMD‐6FFF technique achieved statistically significantly better results than others. (R_50%_ = 0.004; = 0.04; < 0.001 for R_50%_ and *P* = 0.009; = 0.01; < 0.001; < 0.001 for D_2 cm_). DCA‐DMD‐6FFF technique improved R50% and D2 cm values 4%, 8%, 4.5%, 10% and 8.5%, 7%, 7.7% and 9%, respectively, for the other 4 techniques. Normalized graphs of R_50%_ and D_2 cm_ are shown in Fig. 4. In this graph, it is shown that DCA‐DMD‐6FFF technique is superior to other techniques in terms of intermediate dose spillage parameters.

All techniques were compared in terms of mean HI. Mean HI was 0.284 ± 0.06 for DCA‐DMD‐6FFF, 0.276 ± 0.07 for VMAT‐6FFF (*P* = 0.57), 0.299 ± 0.06 for VMAT‐10FFF (*P* = 0.46), 0.334 ± 0.07 for DCA 6FFF (*P* = 0.001), 0.371 ± 0.05 for DCA‐10FFF (*P* < 0.001). There was no statistical significant difference between DCA‐DMD‐6FFF and VMAT techniques. Conversely, there was a statistically significant difference between DCA‐DMD‐6FFF and conventional DCA plans in terms of HI. Furthermore, a linear significant negative correlation was found (*P* = −0.472) between HI and volume of PTV for DCA‐DMD‐6FFF plans. (Data not shown)

**Figure 5:**
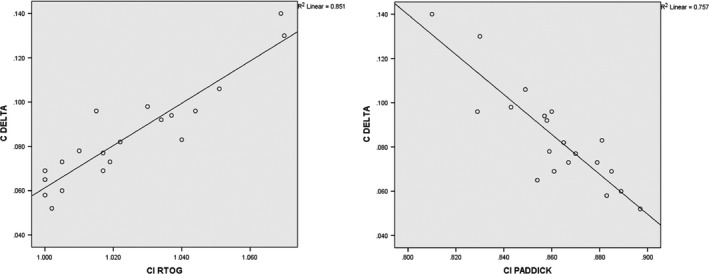
Correlation of CΔ with CI and CI_P__addick_ indexes for DCA‐DMD 6FFF plan.

**Figure 6:**
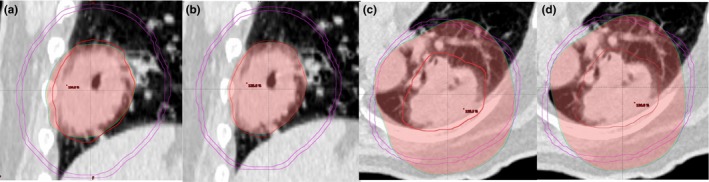
Comparison of VMAT and DCA‐DMD techniques in terms of high and intermediate dose spillage regions (HDS and IDS) for R_100%_ and R_50%_ volumes. (a) VMAT HDS region of R_100%_ (b) DCA‐DMD HDS region of R_100%_ (c) VMAT IDS region of R_50%_ (d) DCA‐DMD IDS region of R_50%_. PTV volume is 123 cc with red color and purple circle is the ring of 2 cm away in all directions from PTV.

CΔ, which is an indicator for the exposure of healthy tissues was also significantly improved by DCA‐DMD‐6FFF plans with respect to others (*P* = 0.002; = 0.001; = 0.001; < 0.001). Mean CΔ was 0.085 ± 0.02 [0.052–0.140] for DCA‐DMD‐6FFF plan and 0.120 ± 0.06 [0.056–0.311] for VMAT‐6FFF. Furthermore, strong positive and negative correlations were found between CΔ with CI and CI_Paddick_ at the level of 0.01 (*P* = 0.873, *P* = −0.809, respectively) which are illustrated in Fig. 5. Dose conformity surrounding PTV showed a strong correlation with dose gradient in the penumbra region. DCA with DMD seemed to improve dose gradient in penumbra region and obtain results comparable to VMAT technique in the vicinity of high dose spillage and intermediate dose spillage regions, as shown in Fig. 6.

As is well known, MUs will be significantly higher for VMAT treatment plans compared to the other techniques. Mean MUs were found to be 2254, 2323, 2132 MU for DCA‐DMD‐6FFF, DCA‐6FFF and 10 FFF, respectively, in this study. Mean MU values were 3250 and 2994 MU for 6FFF and 10FFF of VMAT plans. DCA‐DMD‐6FFF method reduced MUs 44% and 33% percent with respect to VMAT‐6FFF and 10FFF, respectively, (*P* < 0.001; *P* < 0.001) without sacrificing dose conformity.

When all techniques were compared in terms of OAR doses, results showed that the lowest V_20_, V_2.5_, and MLD values were achieved with VMAT. Nevertheless, there was no statistically significant difference between techniques for V_20_ values. (VMAT‐6FFF; 4.03%, DCA‐DMD‐6FFF; 4.19%, DCA‐6FFF; 4.36%, and DCA‐10FFF; 4.46% plans (*P* = 0.88; = 0.62; = 0.079, respectively. V_2.5_ and MLD values were statistically more significant with VMAT‐6FFF (23.83% and 3.42 Gy, respectively) compared to DCA‐DMD‐6FFF (27.88% and 3.72 Gy, respectively), DCA‐6FFF (26.23% and 3.59 Gy, respectively) and DCA‐10FFF (27.41% and 3.72 Gy, respectively) plans. (*P* < 0.001; = 0.003; < 0.001, respectively, for V_2.5_ and *P* < .001; = 0.002; < 0.001), respectively, for MLD).

## 4 DISCUSSION

SBRT has been shown to be a precise and efficient dose delivery method for early stage lung cancer. Still, there is significant variability in terms of treatment techniques among institutions worldwide.^6^, ^7^, ^8^, ^9^, ^35^ Historically, static 3DC treatment was one of the first techniques used in lung SBRT.^1^ Advances in technology, however, have largely replaced 3DC technique with more complex, advanced, and fast modulation techniques such as IMRT, VMAT, and VMAT with FFF photon beams.^11^, ^12^, ^13^, ^14^, ^15^, ^16^, ^17^, ^18^, ^23^, ^24^, ^27^, ^35^ Although the conformity obtained with IMRT is similar to VMAT, delivery time of coplanar and noncoplanar IMRT fields can be 2.6 to 3.7 times longer than VMAT plans.^23^ Currently, VMAT can be considered as an optimal solution with respect to the cost of delivery time.

FFF photon beams permit high dose per pulse through higher dose rate delivery with respect to photon beams obtained with flattening filter. Vassiliev et al.^27^ were the first to report on the physical feasibility for prototype FFF beams modified from a Clinac for early stage NSCLC. They reported a better dose distribution and reduced treatment time with FFF beams. Hrbacek et al.^22^ also stated the FFF beams yielded dose distributions similar to flattened beams with significantly reduced treatment delivery time. Viellevigne et al. also showed the dosimetric gain and efficiency advantages of FFF beams over FF for different sizes of PTV from 1.52 cc to 445.24 cc on virtual phantom, lung and liver.^20^ Thus, a combination of FFF with DCA or VMAT could give optimal SBRT treatment delivery.^20^, ^25^, ^26^

Several studies concluded that VMAT with or without FFF had superior dosimetric conformity parameters when compared to other treatment techniques.^17^, ^18^, ^23^, ^25^, ^26^, ^27^ This advantage came from fluence modulation optimization, with the price of longer treatment planning time and complex quality assurance procedures. Results of this study also have shown that VMAT groups achieve superior dosimetric conformity parameters when compared to conventional DCA groups (Table 3). In the subgroup of FFF energy analysis, VMAT‐6FFF results were superior to VMAT‐10FFF, conventional DCA‐6FFF and 10FFF in terms of CI, CI_Paddick_, R_50%_, D_2 cm_, and CΔ. These results are in agreement with previous studies.^17^, ^18^, ^20^, ^26^, ^35^

However, even though VMAT dosimetric results are superior to conventional DCA, DCA techniques with modifications can give better results than standard 3DC, and may be similar to or better than VMAT plans. Several publications have previously been made on the topic of modified DCA planning.^28^, ^29^, ^30^, ^31^, ^32^ Ross et al. used a modified DCA by expanding PTV volumes 1 or 3 slices more in the superior‐inferior directions only for 20 NSCLC cases.^28^ The isocenter was placed at the lateral midpoint of couch and vertical midpoint of patient in order to avoid collision during arc rotation. Modified DCA plans improved the CI, CI_Paddick_, R_50%_, D_2 cm_ significantly compared to noncoplanar beam. Shi et al. reported their clinical experience for implementation of modified DCA technique for lung and liver SBRT.^29^, ^30^ They also showed that modified DCA was useful and easy to implement. Kim et al. offered a negative margin technique (NMT) in order to improve conventional DCA dose conformation by applying negative margins to PTV in radial directions.^32^ They compared NMT conformal arc plans with zero margin DCA plans for 5 lung cases with 20.5–52.3 cc sizes. NMT plans generated better conformation indices compared to standard DCA plans. Ogura et al. reported modification of PTV to optimize dose distribution in DCA plans for large metastatic brain tumors^31^ by manually fitting modified PTV to the marginal isodose line. Planning was reperformed in iPlan (v 4.5.1, Brainlab, Feldkirchen, Germany) in this study and 24 metastatic brain tumors > 2 cm were planned. DCA plans with modified PTV showed better conformity than nonmodified PTV DCA plans. Nonetheless, the methods used in these previous studies were different from the technique used in our study. In our study, a DCA‐DMD method with manual positive or negative deformation of PTV slice by slice in all required directions with respect to hot and cold spot volumes around PTV was used. This method significantly improved dosimetric parameters compared to VMAT and conventional DCA techniques in terms of CI, CI_Paddick_, R_50%_, D_2 cm_, HI, and CΔ parameters.

Moreover, DCA technique uses MLC‐shaped open fields at control point of beam eye view instead of MLC modulation. While dose rate is changed dynamically during beam on in VMAT treatments, maximum constant dose rate can be delivered in DCA.^21^ As a result, DCA technique generates lesser MU and shorter delivery time than modulated techniques. In this study, it has been shown that DCA treatments may result in 44% and 37% shorter beam on time than VMAT‐6FFF and VMAT‐10FFF techniques, respectively.

When OAR doses were compared for different techniques, it was observed that there were better V_20_, V_2.5_, and MLD values for VMAT‐6FFF. Optimization and exclusion of uninvolved contralateral lung arc sector appears to reduce the parameters significantly for VMAT technique. These findings were supported by the reportings of Zhang et al.^17^ and Navarria et al.^26^. However, the results of the Ong et al.^18^ study have shown contrary results. RTOG 0915 study guideline recommends using minimum 340^°^ arc sectors for coplanar and noncoplanar DCA techniques in order to create better coverage.^33^ Implementation of these recommendations results in increases in V_20_, V_2.5_, and MLD values as expected for DCA.

Reducing delivery time without sacrificing quality of plans is an important goal for departments. This reduction of delivery time necessarily leads to a benefit in terms of cost effectiveness. The new method of DCA introduced in this study makes DCA a simple, fast, and reliable SBRT technique. However, the DCA‐DMD technique is dependent on the trial and error method. This method requires delineation and deformation of a new PTV for dose calculation, which can be time consuming. Nevertheless, this time would seem to be less than the optimization and quality assurance process of VMAT.

## 5 CONCLUSIONS

This study indicates that DCA plans can be improved by using the DMD method. This method overcomes the problems of conventional DCA technique such as hot spot doses adjacent to normal tissue, nonconformal coverage around PTV and hotspot shift out of PTV. Furthermore, DCA with DMD methods lead to similar, if not better, results in terms of dosimetric parameters in comparison to VMAT. It is strongly believed that DCA‐DMD is an efficient and cost‐effective technique for SBRT plans.

## CONFLICT OF INTEREST

The authors have no conflicts of interest.
